# Biochemical, clinical and genetic characteristics in adults with persistent hypophosphatasaemia; Data from an endocrinological outpatient clinic in Denmark

**DOI:** 10.1016/j.bonr.2021.101101

**Published:** 2021-06-28

**Authors:** Nicola Hepp, Anja Lisbeth Frederiksen, Morten Duno, Jakob Præst Holm, Niklas Rye Jørgensen, Jens-Erik Beck Jensen

**Affiliations:** a Dept. of Endocrinology, Hvidovre University Hospital Copenhagen, Kettegaard Alle 30, 2650 Hvidovre, Denmark; b Dept. of Clinical Genetics, Aalborg University Hospital, Ladegaardsgade 5, 9000 Aalborg C, Denmark; c Dept. of Clinical Research, Aalborg University, Fredrik Bajers Vej 7K, 9220 Aalborg Ø, Denmark; d Dept. of Clinical Genetics, University Hospital Copenhagen Rigshospitalet, Blegdamsvej 9, 2100 Copenhagen, Denmark; e Department of Endocrinology, Copenhagen University Hospital Herlev, Borgmester Ib Juuls Vej 1, 2730 Herlev, Denmark; f Dept. of Clinical Biochemistry, Rigshospitalet, Valdemar Hansens Vej 13, 2600 Glostrup, Denmark; g Department of Clinical Medicine, Faculty of Health and Medical Sciences, University of Copenhagen, Blegdamsvej 3 B, 2200 Copenhagen, Denmark

**Keywords:** Alkaline phosphatase, *ALPL*, Hypophosphatasia, Osteoporosis, Bisphosphonates

## Abstract

**Background:**

Hypophosphatasia (HPP) is an inborn disease caused by pathogenic variants in *ALPL.* Low levels of alkaline phosphatase (ALP) are a biochemical hallmark of the disease. Scarce knowledge about the prevalence of HPP in Scandinavia exists, and the variable clinical presentations make diagnostics challenging. The aim of this study was to investigate the prevalence of *ALPL* variants as well as the clinical and biochemical features among adults with endocrinological diagnoses and persistent hypophosphatasaemia.

**Methods:**

A biochemical database containing ALP measurements of 26,121 individuals was reviewed to identify adults above 18 years of age with persistently low levels of ALP beneath range (≤ 35 ± 2.7 U/L). *ALPL* genetic testing, biochemical evaluations and assessment of clinical features by a systematic questionnaire among included patients, were performed.

**Results:**

Among 24 participants, thirteen subjects (54.2%) revealed a disease-causing variant in *ALPL* and reported mild clinical features of HPP, of which musculoskeletal pain was the most frequently reported (*n* = 9). The variant c. 571G > A; p.(Glu191Lys) was identified in six subjects, and an unreported missense variant (c.1019A > C; p.(His340Pro)) as well as a deletion of exon 2 were detected by genetic screening. Biochemical analyses showed no significant differences in ALP (*p* = 0.059), the bone specific alkaline phosphatase (BALP) (*p* = 0.056) and pyridoxal-5′-phosphate (PLP) (*p* = 0.085) between patients with an *ALPL* variant and negative genetic screening. Patients with a variant in *ALPL* had significantly higher PLP levels than healthy controls (*p* = 0.002). We observed normal ALP activity in some patients classified as mild HPP, and slightly increased levels of PLP in two subjects with normal genetic screening and four healthy controls. Among 51 patients with persistent hypophosphatasaemia, fifteen subjects (29.4%) received antiresorptive treatment. Two patients with unrecognized HPP were treated with bisphosphonates and did not show complications due to the treatment.

**Conclusions:**

Pathogenic variants in *ALPL* are common among patients with endocrinological diagnoses and low ALP. Regarding diagnostics, genetic testing is necessary to identify mild HPP due to fluctuating biochemical findings. Antiresorptive treatment is a frequent reason for hypophosphatasaemia and effects of these agents in adults with a variant in *ALPL* and osteoporosis remain unclear and require further studies.

## Highlights

• Mild HPP is overlooked among adults with endocrinological diagnoses.
• Biochemical hallmarks of HPP can fluctuate to normal among mild forms.
• Genetic screening of *ALPL* including MLPA is necessary to identify mild HPP.
• Musculoskeletal pain is the most common symptom in adult HPP.
• Effects of bisphosphonates in mild HPP need further studies.

## 1 Introduction

Hypophosphatasia (HPP), an inherited metabolic disease characterized by deficiency of the enzyme alkaline phosphatase (ALP) is caused by pathogenic variants in the Tissue Nonspecific Alkaline Phosphatase (TNSALP) encoding gene (*ALPL)* (Millán and Whyte, 2016; Weiss et al., 1988)*.*

The HPP presents a wide clinical spectrum including both severe recessive inherited prenatal and dominant inherited milder forms, diagnosed in childhood and adults caused by homozygous/compound heterozygous or heterozygous pathogenic *ALPL* variants, respectively (Hofmann et al., 2013). In adults, HPP may debut as fragility fractures with delayed healing and dental complications including premature loss of permanent teeth and abnormal formation of cementum and enamel (Berkseth et al., 2013; Mori et al., 2016; Schmidt et al., 2017). Furthermore, the patients report unspecific symptoms including muscle weakness, impaired daily physical activity and chronic pain related to muscles, joints and bones (Högler et al., 2019; Schmidt et al., 2017; Weber et al., 2016). The maximal prevalence of mild HPP has recently been estimated to be 1/508 in the European population (Mornet et al., 2020). In contrast, only few patients have until now been diagnosed with HPP in Denmark.

Persistently low levels of ALP represents a characteristically biochemical finding in HPP and pyridoxal-5′-phosphate (PLP), a substrate of TNSALP can be elevated (Whyte, 2017) while identification of pathogenic variant(s) in *ALPL* verifies the clinical diagnosis (Mentrup et al., 2017).

Milder HPP may however, be unrecognized or misdiagnosed because of the non-specific symptoms or unawareness of low ALP (Högler et al., 2019; Maman et al., 2016). Due to a history of low energy fractures or/and low bone mineral density (BMD), patients with unrecognized HPP are in risk to be diagnosed with osteoporosis. Case reports indicate that bisphosphonates (BPs), the most common used drug for osteoporosis, may increase fracture risk and provoke atypical femoral fractures (AFFs) in patients with osteoporosis and unrecognized HPP (Sum et al., 2013; Malabu et al., 2019; Sutton et al., 2012). These reports have led to the assumption, that BPs are contraindicated in HPP (Marini and Brandi, 2017; Shapiro and Lewiecki, 2017).

In addition, BPs and other antiresorptive drugs suppress bone turnover and may decrease levels of ALP and the bone-specific ALP (BALP), an isoform of ALP and marker for bone formation used to monitor antiresorptive treatment (Coburn et al., 1998; Morey et al., 2018; Sarioglu et al., 2006). Consequently, the interpretation of low ALP and thus the recognition of HPP remains challenging in patients with secondary reasons for low ALP including antiresorptive treatment, and little is known about the prevalence of HPP among patients with osteoporosis (Alonso et al., 2020).

Applying biochemical, genetic and clinical investigations, we aimed to identify adults with unrecognized HPP among patients with persistently low ALP and endocrinological diagnoses including osteoporosis. Secondary, we compared clinical and biochemical features in the subjects with genetically verified HPP to the subjects with normal genetic screening.

## 2 Methods

### 2.1 Study population and study design

This cross-sectional study was based on a biochemical database, including ALP measurements registered between February 2004 and 2016, at the Department of Endocrinology, Hvidovre University Hospital Copenhagen (Denmark). The database was reviewed to identify individuals with persistent hypophosphatasaemia and the normal range for ALP in adults (35–105 U/L; ≥18 years of age) has not been changed in the defined period.

Individuals above 18 years of age at the first ALP measurement and at least two ALP measurements ≤35 U/L and none ≥35 (± 2.7) U/L, were invited to participate in the study by letter. Patients, who returned a signed reply letter and agreed to receive further information were contacted by telephone. The study included an information conversation, a screening visit and a main study visit, where we took blood samples for genetic screening, performed a physical examination and a questionnaire survey. Results of genetic screening where given to the participants at a closing conversation with genetic counselling.

Exclusion criteria included renal failure (eGFR ≤40 mL/min), current malignant disorders, Cushing's disease, manifest and untreated hypothyroidism (T4 below normal range) ≥ 6 months (at screening) and pregnancy. All participants provided oral and written informed consent. The study was carried out in accordance with the Helsinki Declaration and was approved by the regional ethical committee (Journal-nr.: H-20011193) and the data protection agency (j.nr.: AHH-2017-076; I-Suite nr.: 05857).

### 2.2 Biochemical parameters

Measurements of ALP activity were performed on a Vitros 5.1 analyzer (Ortho Clinical Diagnostics, Inc., USA) from February 2004 to May 2010. From May 2010 to date, ALP has been analyzed on the COBAS 8000 from Roche Diagnostics GmbH, Mannheim, Germany. Internal laboratory data showed an acceptable comparison between the two systems.

After overnight fasting, blood samples for biochemical analysis were taken at the screening visit between 8.00 and 10.00 a.m. Participants had paused all supplements, containing vitamin B6 and calcium, for at least two weeks. PLP was analyzed by high pressure liquid chromatography (HPLC) analysis (Chromosystems Instruments and Chemical GmbH, Germany). Batch analyses of PLP were conducted for the *ALPL* groups and the control groups, respectively. BALP was batch analyzed and measured by an iSYS automated analyzer using an immune-based chemiluminescence assay (Immunodiagnostic Systems, Tyne and Wear, UK).

### 2.3 Genetic analyses

Sanger sequencing was performed to screen the coding and intron flanking sequences of *ALPL* (NM_000478.5). DNA samples, in which no variant was identified by Sanger sequencing, were subsequently analyzed by Multiplex Ligation-dependent Probe Amplification (MLPA) to identify any larger deletions or duplications. The MLPA was conducted according to the manufacturer's instructions (SALSA MLPA Probemix P484 ALPL, MRC Holland, Netherlands). Primers and PCR conditions are available upon reasonable request.

### 2.4 Clinical assessments

Information about clinical features, co-morbidities and medical treatment was assessed by a questionnaire survey and reviewing the clinical journals and electronical medication records. The questionnaire was validated and contained information about fracture history, pain pattern, physical activity and dental status. Responses on physical activity and pain frequency were graded on a Likert scale. Questions about pain duration and dental status had drop down answers graded as different age and time periods. In addition, information about fractures was obtained from the clinical records and documented X-rays. Fractures were classified as low- or high energy fractures. Muscle weakness was specified to be manifest, when the participant reported to have muscle weakness as well as limited physical activity due to muscle weakness “most of the time” or “worsening over time” (Likert scale: no; yes, sometimes; yes, most of the time; worsening over time). Pain in joints, bones and muscles was defined as chronic, when stated to be present “frequently” or “almost all the time” (Likert scale: never; seldom; sometimes; frequently; almost all the time) as well as being present for at least one year (range: ≤ 1 year; 1–6 months; 0.5–1 year; 1–2 years; 2–5 years). Premature tooth loss was defined as loss of baby teeth ≤3 years of age (range: ≤ 3 years of age; ≤ 5 years of age; 6–10 years of age; cannot remember). The diagnosis of early loss of permanent teeth (not related to trauma) was given to patients, who have lost six or more teeth (range: 1–2 teeth; 3–4 teeth; 5–6 teeth; ≥ 6 teeth) and had the first tooth loss before the age of 30 years (open question). Dual energy X-ray absorptiometry (DXA) of the lumbar spine and the hip (Hologic Inc., MA, USA, Horizon™ QDR™ Series) as well as a vertebral fracture assessment (VFA) were performed. The diagnosis of osteoporosis was given to patients with a low energy fracture of the hip or vertebrae (≥ 20% decrease in vertebral height) and/or low BMD (T-score ≤ −2.5) in the lumbar spine or hip (total hip or femoral neck).

### 2.5 Statistical analysis

The prevalence estimation of subjects with a variant in *ALPL* among adults with persistent hypophosphatasaemia and the comparison of clinical manifestations between the *ALPL+* and *ALPL-* group were based on descriptive analysis. Categorial variables were described as an absolute number and relative percentage, and continuous variables as median and interquartile range (IQR). To compare median of demographic and biochemical results between the groups, the Wilcoxon's rank-sum test was applied, as data were not normally distributed. *P* values <0.05 were defined as statistically significant. Statistical analyses were performed using the R studio statistical software (version 3.6.1).

## 3 Results

### 3.1 Population and genetical findings

**Fig. 1:**
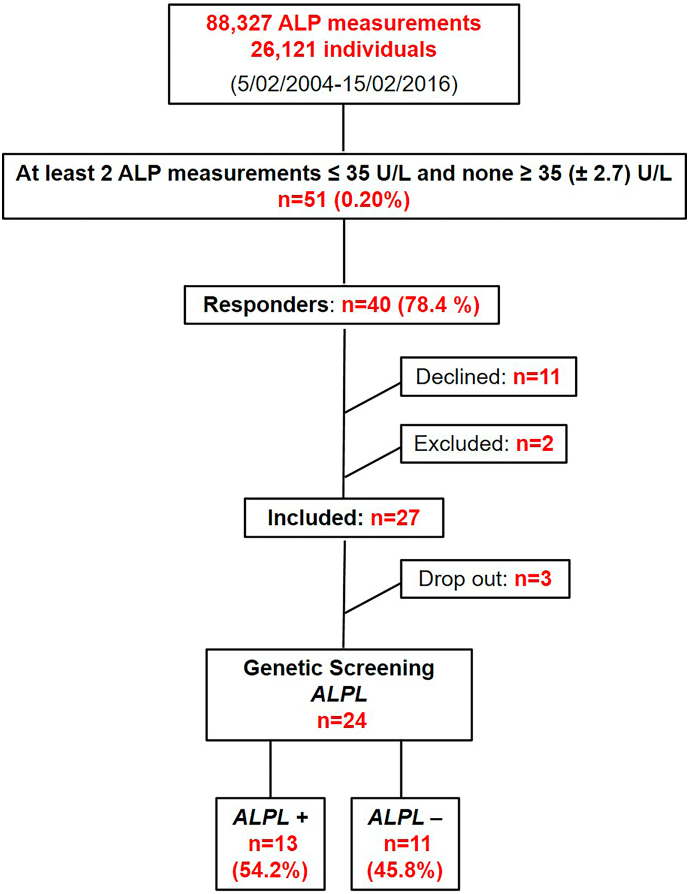
Flowchart visualising the inclusion and recruitment process of the study**.** ALP = alkaline phosphatase. *ALPL+* = participants in whom a variant in *ALPL* was identified. *ALPL-* = individuals with normal genetic screening.

The reviewed laboratory database included 88,327 ALP measurements from 26,121 individuals. Persistent hypophosphatasaemia was found in 51 patients (0.20%). Among the 51 patients with persistent hypophosphatasaemia, 25 (49%) were referred to our outpatient clinic due to screening or treatment for osteoporosis or other bone diseases. Two patients (4%) already died and the reason for referral could not be investigated. Further reasons for referral included hypothyroidism (13.7%, *n* = 7), obesity (7.8%, *n* = 4) and other reasons including screening for disorders of sexual hormones and the adrenal gland, Struma, participation in research projects as well as other unspecific symptoms (25.5%, *n* = 13). Out of 27 included patients, 24 adults underwent genetic screening. Sanger sequencing of *ALPL* revealed a pathogenic (P) or likely pathogenic (LP) heterozygous missense variant in 12 participants. In one patient, a heterozygous deletion of exon 2 was identified by MLPA. The prevalence of adults with a variant in *ALPL* among patients with persistently low levels of ALP was 54.2% *(ALPL+ group)* (see Fig. 1).

All subjects in the *ALPL*+ group were heterozygous for a single variant in *ALPL.* Upon all detected variants, 10 were classified as pathogenic (P) and have previously been reported in patients with HPP (see Table 1). One novel missense variant was classified as likely pathogenic (LP) and the exon 2 deletion as pathogenic. All genetic variant classifications were performed according to the ACMG guidelines (Richards et al., 2015). The recurrent c. 571G > A; p.(Glu191Lys) variant was identified in six participants. Among subjects with positive genetic screening, one patient with HPP and pycnodysostosis (ALP 027) has been published previously and was excluded from further analysis (Hepp et al., 2019). All the genetic findings are summarized in Table 1.

**Table 1:** Variants in *ALPL*, identified in the *ALPL+* group.

Subject	*ALPL* exon	Variant (cDNA)	Variant (amino acid)	Classification	References
ALP 001	11	c.1250A > G	p.(Asn417Ser)	P	Sergi et al., 2001; del Angel et al., 2020
ALP 003	6	c. 571G > A	p.(Glu191Lys)	P	Henthorn et al., 1992; del Angel et al., 2020
ALP 004	10	c.1019A > C	p.(His340Pro)	LP	Unpublished
ALP 005	3	c.98C > T	p.(Ala33Val)	P	Henthorn et al., 1992; Reis et al., 2020
ALP 008	6	c. 571G > A	p.(Glu191Lys)	P	Henthorn et al., 1992; del Angel et al., 2020
ALP 011	6	c. 571G > A	p.(Glu191Lys)	P	Henthorn et al., 1992; del Angel et al., 2020
ALP 014	6	c.526G > A	p.(Ala176Thr)	P	Taillandier et al., 2000; del Angel et al., 2020
ALP 018	2	deletion	p.(?)	P	Unpublished
ALP 019	6	c. 571G > A	p.(Glu191Lys)	P	Henthorn et al., 1992; del Angel et al., 2020
ALP 020	6	c. 571G > A	p.(Glu191Lys)	P	Henthorn et al., 1992; del Angel et al., 2020
ALP 021	6	c. 571G > A	p.(Glu191Lys)	P	Henthorn et al., 1992; del Angel et al., 2020
ALP 026	12	c.1366G > A	p.(Gly456Arg)	P	Ozono et al., 1996; del Angel et al., 2020
ALP 027	12	c.1487A > G,	p.(His496Arg)	LP	Hepp et al., 2019

Pathogenicity classifications are based on the ACMG guidelines (Richards et al., 2015). P = pathogenic, LP = likely pathogenic.

### 3.2 Biochemical results

**Fig. 2:**
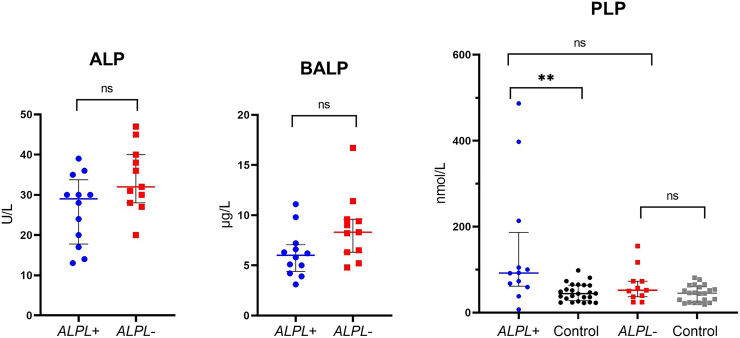
Individual measurements and median of alkaline phosphatase (ALP), the bone specific ALP (BALP) and pyridoxal-5′-phosphate (PLP) in the *ALPL*+ (*n* = 12) and *ALPL*- (*n* = 11) group at the screening visit. PLP values of both groups were compared with healthy controls, matched 1:2 (Control to *ALPL*+, *n* = 24; Control to *ALPL*-, *n* = 22). Reference intervals: ALP (18–115 years): 35–105 U/L; BALP M (40–125 years): 7.5–25.1 μg/L, W (25–30 years): 5.9–30.0 μg/L, W (30–50 years): 6.5–21.1 μg/L, W (50–125 years): 8.3–29.4 μg/L; PLP (15–73 nmol/L). ns = not significant, ** < 0.01, M = Men, W = Women.

Patients in the *ALPL+* group had though not significant, a trend towards lower levels of ALP (median [IQR]) (29.0 [19.3–31.3] U/L vs. 32.0 [29.0–39.0] U/L; *p* = 0.059) and BALP (6.0 [4.8–6.8] μg/L vs. 8.3 [6.4–9.5] μg/L; *p* = 0.056), compared with the *ALPL*- group. At screening, ALP levels were in the lower normal range in three patients in the *ALPL+* and in five subjects in the *ALPL-* group. Though not significant, there was a trend to higher PLP (median [IQR]) in patients with pathogenic *ALPL* variant (92.0 [65.6–132.1] nmol/L vs. 52.0 [38.2–73.0] nmol/L; *p* = 0.085). In addition, PLP values of both groups were compared with healthy controls who have had persistently normal ALP measurements (no ALP measurements beneath 45 U/L). Cases of the *ALPL*+ and *ALPL*- group were matched 1:2 with healthy controls for gender and age (±13 years). The *ALPL*+ and the *ALPL*- group did not differ significantly in age compared with the control groups: *ALPL*+ vs. Control (median [IQR]) (51.5 [44.3–56.3] years vs. 50.0 [44.5–57.0] years; *p* = 0.848); *ALPL*- vs. Control (median [IQR]) (50.0 [43.0–60.5] years vs. 49.5 [40.0–55.8] years; *p* = 0.818). The *ALPL*+ group had significantly higher PLP values than controls (median [IQR]) (92.0 [65.6–132.1] nmol/L vs. 43.8 [29.4–58.5] nmol/L; *p* = 0.002). In comparison, PLP values did not differ significantly between the *ALPL*- group and controls (median [IQR]) (52.0 [38.2–73.0] nmol/L vs. 45.1 [25.1–57.8] nmol/L; *p* = 0.163). ALP, BALP and PLP measurements including reference ranges are visualized in Fig. 2.

Additional biochemical and demographic results and reference intervals are listed in Table 2. In both groups, none had elevated calcium ion and one participant in the *ALPL+* group had increased phosphate. Severe deficiency of magnesium, zinc and D-vitamin as well as manifest hypothyroidism, elevated liver parameters and positive M-component were not present in any subjects at screening.

**Table 2:** Demographic and biochemical parameters of patients in the *ALPL-* and *ALPL+* group.

	*ALPL+* (n = 12)	*ALPL-* (n = 11)
Age (years)	51.5 (44.3–56.3)	50.0 (43.0–60.50)
BMI (kg/m^2^)	26.1 (24.1–30.7)	23.2 (21.7–25.0)
Height (cm)	170.3 (165.7–171.9)	163.8 (160.2–171.9)
Sitting height (cm)	90.92 (88.33–92.86)	86.00 (84.85–90.47)
Head circumference (cm)	55.88 (55.48–56.25)	55.50 (54.60–56.25)
Gender (f/m)	(11/1)	(8/3)
Calcium ion (1.18–1.32 mmol/L)	1.22 (1.20–1.23)	1.22 (1.20–1.23)
Phosphate (0.76–1.41 mmol/L)	1.17 (1.00–1.27)	1.03 (0.91–1.12)
Magnesium (0.71–0.94 mmol/L)	0.84 (0.77–0.88)	0.82 (0.81–0.86)
Zinc (10–19 μmol/L)	11 (10−11)	11 (10−12)
D-vitamin (≥ 50 nmol/L)	78 (60–130)	75 (53–151)
ALAT (10–45 U/L)	21 (17–25)	23 (20–26)
TSH (0.65–4.80 *10–^3^ IU/L)	1.90 (1.63–2.60)	1.57 (1.13–1.85)
PTH (1.1–7.1 pmol/L)	5.0 (3.8–5.5)	5.2 (4.0–5.7)

Values are median (IQR) and did not differ statistically significant between the two groups.

### 3.3 Clinical manifestations

All patients in the *ALPL*+ group reported at least one symptom associated to HPP. Most discovered clinical signs included chronic pain in muscles, bones and joints (*n* = 9/12) as well as muscle weakness (*n* = 5/12). Four subjects experienced premature loss of deciduous or adult teeth. We classified patients as mild HPP if they fulfilled all of the following criteria: (1) persistently low ALP (at least 2 measurements ≤35 U/L); (2) heterozygous or compound heterozygous for a pathogenic or likely pathogenic variant in *ALPL*, following the ACGM guidelines; (3) at least two of the following symptoms: (A) history of a low energy fracture, (B) premature loss of baby or permanent teeth, (C) chronic musculoskeletal pain, (D) muscle weakness. Nine out of twelve subjects were classified as mild symptomatic HPP patients.

Opposite, manifest muscle weakness and premature tooth loss were not present in the *ALPL-* group and chronic musculoskeletal pain was less frequent (*n* = 2/11). Most patients in the *ALPL+* (*n* = 9/12) *and ALPL-* group (*n* = 7/11) stated to have had a fracture. In the *ALPL-* group most fractures were associated to osteoporosis. Furthermore, possible secondary reasons for hypophosphatasaemia including antiresorptive treatment and previously manifest hypothyroidism were more present in the *ALPL-* group (8/11), compared with the *ALPL+* group (4/12).

### 3.4 Patients with previous or current antiresorptive treatment

In our cohort, 15 out of 51 individuals (29.4%) with repeatedly low levels of ALP had currently or previously received antiresorptive agents. Fifty percent of these patients (n = 7) were examined by genetic screening and two patients revealed a pathogenic variant in *ALPL* (ALP 004 and ALP 026). In these two patients (postmenopausal women), ALP activity was ≤20 U/L (14; 17 U/L) and they had the highest levels of PLP (397.5; 486.7 nmol/L), but the lowest BALP measurements (3.1; 3.9 μg/L) among all participants. ALP 004 has received Alendronate for four years due to low BMD and did not develop any vertebral nor peripheral fractures. The other patient, a woman with low BMD, anorexia and multiple fractures, previously received Alendronate and Zoledronic acid. Four out of eight fractures occurred after BP treatment and this patient experienced most fractures among all participants. None of the two patients developed AFFs or femoral pseudofractures, proved by X-ray of the hip and femoral bones. All clinical features, co-morbidities and information about antiresorptive treatment are listed in Table 3 (see Supplementary Material).

## 4 Discussion

To the best of our knowledge, this is the first study, to investigate the occurrence of unrecognized HPP among patients with endocrinological diagnoses and persistently low levels of ALP. The prevalence of persistent hypophosphatasaemia was 0.20% in our study, which is in accordance with previous studies investigating the prevalence of hypophosphatasaemia among adults in Spain (Tornero et al., 2020) and France (Maman et al., 2016), respectively. In comparison, these studies addressed ALP measurements in a larger clinical context and had a much higher sample size. In addition, one study reviewing ALP values from patients referred to an osteoporosis clinic, reported a higher prevalence of persistent hypophosphatasaemia (0.49%) (Alonso et al., 2020). The estimated prevalence of subjects with a disease-causing variant in *ALPL* (54.2%) was slightly higher in our study, compared with other European studies (50% and 47%) (Riancho-Zarrabeitia et al., 2016; Tornero et al., 2020). On the other hand, trials which choose a lower cut off for ALP measurements, achieved a higher prevalence of subjects in whom a variant in *ALPL* is identified (McKiernan et al., 2017). Contrary to these studies, the most common pathogenic *ALPL* variant in our cohort was c. 571G > A; p.(Glu191Lys), which is in accordance with the assumption that c. 571G > A; p.(Glu191Lys) is the predominant variant in Northern Europe (Mornet et al., 2020). In addition, our results support this is a founder variant in the Nordic countries (Hérasse et al., 2002; Mornet et al., 2020). Consequently, our data show that mild HPP is an overlooked condition among patients with endocrinological diagnoses, and the prevalence of HPP in Denmark is compatible with that seen in other European countries.

In daily clinical practice, it is a diagnostic challenge to distinguish hypophosphatasaemia caused by HPP from secondary causes to low ALP including endocrinological conditions such as hypothyroidism and BP treated osteoporosis (McKiernan et al., 2017). Of notice, three patients of the *ALPL*- and two of the *ALPL*+ group previously had profound hypothyroidism, which may have contributed to impaired ALP activity. ALP levels in these patients did though remain low after treatment with thyroid hormones and biochemically normalized thyroid parameters (measured at screening). Long term suppression of ALP values in these patients may be explained by a supressed bone turnover and a higher sensitivity for this condition due to hypothyroidism, which may have led to a prolonged recovery of ALP activity.

Here, we compared biochemical and clinicals features between the *ALPL+* and *ALPL-* group to investigate, if biochemical and clinical differences may be useful to improve diagnostics. Our results show that patients with mild HPP can have ALP levels in the lower normal range (see Fig. 2). In addition, a recently published study, presenting a large cohort of patients suspected for HPP, reported that low normal ALP activity can occur in mild symptomatic HPP patients (Jandl et al., 2020). However, PLP is assumed to be a more specific and sensitive marker to detect HPP (Riancho-Zarrabeitia et al., 2016). In our study, only two patients in the *ALPL-* group had elevated PLP. One of these patients had normal PLP in a clinical control after participating in the study. Unfortunately, we were not able to measure PLP again in the other patient. PLP analysis was performed twice with the same blood samples and we did not see significant changes between the two measurements. The elevated PLP levels in these patients could be explained by certain nutrition intake (supplements were paused for two weeks) or secondary induced ALP impairment. Opposite, increased PLP in patients with normal genetic screening was not reported by Riancho et al. (Riancho-Zarrabeitia et al., 2016). In addition, five out of 46 controls had slightly elevated PLP values (73.2–98.1 nmol/L, see Fig. 2). A recently published study investigated possible fluctuations of PLP in adults and established race-, gender- and age-specific reference intervals for PLP, has shown that inflammation and chronic kidney disease can lower PLP levels (Schini et al., 2020). In the *ALPL+* group, most participants had elevated PLP, including one patient with slightly decreased kidney function and inflammation parameters were normal among all participants in this group. Furthermore, PLP was elevated in participants with a variant in *ALPL* and normal ALP activity. Our results indicate that elevated PLP can be useful to detect mild HPP and to distinguish from secondary hypophosphatasaemia, but PLP measurements can fluctuate and can also be slightly elevated in individuals with normal ALP. Due to the variability and the high costs of PLP analysis, we suggest that ALP measurements and genetic screening are adequate to detect mild symptomatic individuals with pathogenic variant(s) in *ALPL*.

Regarding clinical features, all patients in the *ALPL+* group reported a least one clinical sign, which can be related to HPP, and musculoskeletal pain was most strongly associated with a disease-causing variant in *ALPL.* Our data on clinical signs confirm that pain may be the most common symptom and burden in adult HPP (Högler et al., 2019; Weber et al., 2016). A weakness of the present study is that the evaluation of pain, muscle weakness and dental status is based on a questionnaire survey and not on physical examinations. In addition, we did not have paediatric and dental records available to identify possible symptoms of HPP before adulthood. However, we have studied a small cohort of patients with a disease-causing variant in *ALPL* and the clinical signs of these patients are often unspecific. To better understand the clinical impact of ALP impairment in these patients, further studies including a lager sample size and a control group comprised of the general population are needed.

Due to fluctuating biochemical parameters and unspecific clinical symptoms in adults with mild HPP, we assume that genetic screening is an important tool in HPP diagnostics. By Sanger sequencing approximately 95% of all mutations in *ALPL* can be detected (Mornet, 2000; Taillandier et al., 2015). Compared to previous studies (García-Fontana et al., 2019; Maman et al., 2016; Riancho-Zarrabeitia et al., 2016; Tornero et al., 2020), we also included MPLA for detection of deletions and duplications and found one patient with a large deletion of exon 2, indicating that MLPA should be considered in patients with signs of HPP and normal Sanger sequencing.

At our endocrinological outpatient clinic, we evaluate and treat more than 7500 patients with osteoporosis per year. Considering antiresorptive treatment may be contraindicated in HPP, it is of clinical relevance to identify the patients with HPP among the group of osteoporotic subjects. While we could show that antiresorptive treatment is a frequent reason for persistent hypophosphatasaemia, we only found two patients with unrecognized HPP and BP treated osteoporosis. In contrast, a single study of 3285 patients referred to an osteoporosis clinic, reported a higher prevalence of patients with an *ALPL* variant (14 out of 16 patients with hypophosphatasaemia) including four subjects treated with BPs (Alonso et al., 2020). Furthermore, this study could not show a higher fracture rate in these patients compared to a BP treated control group (Alonso et al., 2020) which is in line with our finding.

## 5 Conclusions

Our data show, that unrecognized mild HPP is common among adults with endocrinological diagnosis and low levels of ALP. Clinical results indicate that musculoskeletal pain is the most common symptom, associated with a pathogenic variant in *ALPL*. However, clinical features in adults, heterozygous for a pathogenic variant in *ALPL* are often unspecific and further studies are required to investigate the clinical impact of this condition. Further, genetic screening may be the most important diagnostic tool due to fluctuations in measurements of PLP and ALP in mild affected HPP patients. In our cohort, the co-incidence of BPs treated unrecognized HPP was limited to only two patients who did not show AFFs. However, osteoporosis treatment in patients with HPP remains challenging and further studies are needed to improve knowledge about the effects of BPs in these patients.

The following is the supplementary data related to this article.

Table 3 HPP related symptoms, co-morbidities and information about antiresorptive treatment in the ALPL+ and ALPL- group. Table 3

## Funding

This research was supported by The 10.13039/501100006197A.P. Møller Foundation for the Advancement of Medical Science, Denmark (Journalnr. 17-L-0254).

## CRediT authorship contribution statement

**Nicola Hepp:** Project administration, Conceptualization, Methodology, Investigation, Data curation, Formal analyses, Writing - original draft. **Anja Lisbeth Frederiksen:** Conceptualization, Supervision, Writing - review & editing. **Morten Duno:** Resources, Supervision, Writing - review & editing. **Jakob Præst Holm:** Data curation, Writing - review & editing. **Niklas Rye Jørgensen:** Resources, Writing - review & editing. **Jens-Erik Beck Jensen:** Conceptualization, Methodology, Supervision, Writing - review & editing.

## Declaration of competing interest

**Anja Lisbeth Frederiksen, Morten Duno, and Niklas Rye Jørgensen** have no known competing financial interests or personal relationships that could have appeared to influence the work reported in this paper. **Nicola Hepp** has received research funding from Alexion Pharmaceuticals, Inc. **Jakob Præst Holm** has participated as a sub investigator in studies by Amgen and MSD, and received payment for lectures sponsored by 10.13039/100002429Amgen and LEO Pharma. **Jens-Erik Beck Jensen** is a board member in Eli Lilly, Amgen, Gedeon Richter and UCB, received funding from Eli Lilly and Amgen and consulting fees from UCB, Giliad and Amgen.
