# Supplementary material for: Biochemical, clinical and genetic characteristics in adults with persistent hypophosphatasaemia; Data from an endocrinological outpatient clinic in Denmark
Source: Bone Rep. 2021 Jun 28;15:101101. doi: 10.1016/j.bonr.2021.101101 (PMC8256181; doi:10.1016/j.bonr.2021.101101)
Supplement: Table 3 — HPP related symptoms, co-morbidities and information about antiresorptive treatment in the ALPL+ and ALPL- group. [file mmc1.docx]

Supplementary material

**Table 3** HPP related symptoms, co-morbidities and information about antiresorptive treatment in the *ALPL+* and *ALPL-* group

| **Subject (age)** | **HPP related symptoms** | **Co-morbidities** | **Antiresorptive treatment** | **Reason for referral** |
| --- | --- | --- | --- | --- |
| ***ALPL+* group** | | | | |
| ALP 001  (42 years) | - Stress fracture of the right 5th. metatarsal  - Muscle weakness  - Chronic pain in muscles and knees | - Obesity | - No | - Participated in a research project for osteoporosis screening |
| ALP 003  (72 years) | - Low energy fracture, right elbow  - Muscle weakness  - Chronic pain in muscles, bones, neck, back, hip and knees. | - Parkinson’s disease  - OP for spinal stenosis  - Scoliosis | - No | - Screening for osteoporosis |
| ALP 004  (69 years) | - Chronic pain in bones, hands and back  - Early loss of permanent teeth | - Osteoporosis  - OP for rotator cuff syndrome, right shoulder  - Migraine  - Depression | - Alendronate (4 years) | - Screening and treatment for osteoporosis |
| ALP 005  (52 years) | - Chronic pain in the neck  - Early loss of permanent teeth | - Obesity | - No | - Participated in a research project due to obesity |
| ALP 008  (42 years) | - Low energy fracture, clavicula  - Muscle weakness  - Chronic pain in muscles, shoulders, back and hip | - Spinal stenosis  - Spinal disk herniation  - Hip with Cam deformity  - Hashimoto thyroiditis | - No | - Screening for thyroid disease and disorders of the adrenal gland |
| ALP 011  (69 years) | - Low energy fractures of the right little toe (2x) and the 3. toe | - Osteopenia  - M. Scheuermann  - Asthma | - No | - Screening for osteoporosis |
| ALP 014  (45 years) | -Low energy fracture, right radial head  - Muscle weakness  - Chronic pain in muscles, bones, shoulders, neck, back and right hip | - Gastric bypass  - Arthrosis of the right hip and lumbar spine  - PCO-Syndrome | - No | - Obesity and bariatric surgery |
| ALP 018  (33 years) | - High energy fracture of the right hand, 3. and 4. finger.  - Low energy fracture of a toe, right foot.  - Chronic pain in, right arm, back, right hip, right knee and foot. | - Asthma | - No | - Screening for thyroid disease and disorders of the adrenal gland |
| ALP 019  (52 years) | Premature tooth loss of baby teeth | - Gastric bypass  - Hypercholesterolemia  - Osteopenia | - No | - Obesity and bariatric surgery |
| ALP 020  (51 years) | - Low energy fracture of the right 3. and 5. toe  - Muscle weakness  - Chronic pain in muscles, left arm and hand, back, left knee and right foot | - Hypertension  - Struma  - Spinal stenosis  - Spinal disk herniation  - Hip with Cam deformity | - No | - Struma |
| ALP 021  (52 years) | - High energy fracture of the left little finger  - Chronic pain in muscles, right shoulder, right hand and knees | - Struma  - Hypothyroidism after hemithyroidectomy  - Arthrosis of the knee | - No | - Struma |
| ALP 026  (45 years) | - Pre BPs: low energy fracture of the left humerus,  stress fracture of left 3rd. and 4th. metatarsal, high energy fracture of the acetabulum,  - Post BPs: high energy fracture of the left clavicula, right and left calcaneus and right humerus neck.  - Early loss of permanent teeth | - Anorexia nervosa  - Osteoporosis | - Alendronate (2 years)  - Zoledronic acid  (2 years) | - Screening for osteoporosis |
| ***ALPL-* group** | | | | |
| ALP 002  (47 years) | - Low energy fracture of left clavicula and three toes (left food)  - Single event with polyarthritis (pyrophosphate crystals in joints) | - Hypothyroidism and hypoparathyroidism after total thyroidectomy | - No | - Struma and hypothyroidism |
| ALP 006  (53 years) | - Low energy fracture of right distal fibula and os naviculare  - Stress fracture, right 5th. metatarsal  - Chronic pain in muscles, bones, shoulders, neck, back right arm, hand, hip, knee and foot | - Osteoporosis  - Scoliosis | - Zoledronic acid (1 year) | - Treatment for osteoporosis |
| ALP 007  (68 years) | - High energy fracture of clavicula, hand and little finger | - Sarcoidosis  - Osteopenia | - Alendronate (2 years) | - Screening for osteoporosis due to prednisolone treatment |
| ALP 009  (55 years) | - Low energy fracture of right ankle and left radius | - Subclinical thyrotoxicosis  - Osteoporosis | - Alendronate (2 years)  - Zoledronic acid  (2 years) | - Screening for osteoporosis |
| ALP 010  (66 years) | - None | - Osteopenia (previous) | - No | - Screening for osteoporosis |
| ALP 012  (50 years) | - None | - Osteoporosis | - No | - Screening for osteoporosis |
| ALP 015  (84 years) | - Low energy fracture of the left radius and patella  - Chronic pain in left shoulder, neck, hands, back and feet | - Osteoporosis  - Apoplexia cerebri | - Alendronate (6 months)  - Ibandronic acid (1dose)  - Denosumab (7 years) | - Treatment for osteoporosis |
| ALP 016  (34 years) | - None | - Graves’ disease (previous) |  | - Thyrotoxicosis |
| ALP 017  (45 years) | - Low energy fracture of the left hip  - High energy vertebral fracture Th11 (60%) and high energy fracture of one finger (left hand) | - Osteoporosis  - Migraine | - Ibandronic acid  (3 years)  - Zoledronic acid  (4 years) | - Screening and treatment for osteoporosis |
| ALP 023  (31 years) | - High energy fracture of the left arm | - Hashimoto thyroiditis | - No | - Hypothyroidism |
| ALP 025  (41 years) | - None | - Hashimoto thyroiditis | - No | - Hypothyroidism |
